# The Current State of the Art in Acute Kidney Injury

**DOI:** 10.3389/fped.2020.00070

**Published:** 2020-03-17

**Authors:** Prasad Devarajan

**Affiliations:** Department of Nephrology and Hypertension, Cincinnati Children's Hospital Medical Center, University of Cincinnati College of Medicine, Cincinnati, OH, United States

**Keywords:** acute kidney injury, electronic triggers, clinical scores, biomarkers, anti-fibrotic therapies

## Abstract

Decades of pre-clinical research have revealed biologic pathways that have suggested potential therapies for acute kidney injury (AKI) in experimental models. However, translating these to human AKI has largely yielded disappointing results. Fortunately, recent discoveries in AKI molecular mechanisms are providing new opportunities for early detection and novel interventions. This review identifies technologies that are revealing the exceptionally complex nature of the normal kidney, the remarkable heterogeneity of the AKI syndrome, and the myriad responses of the kidney to AKI. Based on the current state of the art, novel approaches to improve the bench-to-bedside translation of novel discoveries are proposed. These strategies include the use of unbiased approaches to improve our understanding of human AKI, establishment of irrefutable biologic plausibility for proposed biomarkers and therapies, identification of patients at risk for AKI pre-injury using clinical scores and non-invasive biomarkers, initiation of safe, and effective preventive interventions of pre-injury in susceptible patients, identification of patients who may develop AKI post-injury using electronic triggers, clinical scores, and novel biomarkers, employment of sequential biomarkers to initiate appropriate therapies based on knowledge of the underlying pathophysiology, use of new biomarkers as criteria for enrollment in randomized clinical trials, assessing efficacy, and empowering the drug development process, and early initiation of anti-fibrotic therapies. These strategies are immediately actionable and hold tremendous promise for effective bench-to-bedside translation of novel discoveries that will change the current dismal prognosis of human AKI.

## Introduction

Acute kidney injury (AKI) is a growing global epidemic, afflicting about 30% of children in neonatal and pediatric intensive care units and at least 5% of non-critically ill pediatric hospitalizations. Impressive improvements in the clinical care of hospitalized children have inexorably shifted the AKI epidemiology from primary renal diseases toward a consequence of systemic illnesses and their treatments and nephrotoxin exposure. In contrast with AKI in adults, pediatric AKI typically strikes early in the course of a critical illness, but it is more often reversible in the absence of major comorbid complications. However, AKI is independently associated with increased mortality and morbidity, including the development of chronic kidney disease, in all age groups. Several decades of intense pre-clinical and translational research have uncovered biologic pathways and mechanisms that have suggested promising therapeutic approaches in animal models. However, the translational efforts in human AKI have largely yielded disappointing results, and the incidence of and the complications from human AKI remain unacceptably high, with no satisfactory preventive or therapeutic solutions in sight. Fortunately, recent discoveries in AKI molecular mechanisms are shifting old paradigms and providing new approaches for early detection and intervention. This review will focus on the state-of-the-art technologies that are revealing the exceptional complexity of the normal kidney, the remarkable heterogeneity of the AKI syndrome, and the myriad responses of the kidney to AKI. Novel approaches to improve the bench-to-bedside translation of novel discoveries will be proposed—at the present time, these remain largely the author's personal opinions that have not been systematically studied.

## The Unexpected Complexity of The Normal Kidney

Our understanding of the normal kidney has advanced dramatically during the past decade, with the advent of unbiased gene, protein, and metabolome expression analysis, propelled by the enabling technologies of molecular nephrology (1, 2). In particular, single-cell RNA sequencing (scRNA-seq) can now uncover the expression level of every gene in every cell type, enabling the rapid determination of serial gene expression changes in many thousands of cells, identification of previously unknown cell populations, and even novel heterogeneity within a given cell type. For example, the scRNA-seq analysis of the developing collecting duct has newly identified sub-cluster cell types that include principal cells, β-intercalated cells, and other previously unknown cell subtypes (3). Similar studies in the fully developed collecting duct have revealed a novel crosstalk between signal transduction pathways as well as an improved understanding of physiologic regulatory pathways (4). A comprehensive scRNA-seq analysis of adult mouse kidneys has identified novel cell types that remain to be fully characterized (5). An examination of human kidney transplant biopsies has uncovered 16 distinct cell types and novel cell states within endothelial cells as well as pro-inflammatory parenchymal responses in the rejecting kidney (6). Thus, scRNA-seq techniques are identifying new categories of known cell sub-types as well as previously unknown cell types that are improving our understanding of the developing, mature, and diseased kidney at an unprecedented level of detail.

## The Unanticipated Heterogeneity of Acute Kidney Injury

Decades of meticulous clinical phenotyping have taught us that all AKIs are not created equal. As clinicians, we have become adept at recognizing several AKI subtypes, including pre-renal, intrinsic, ischemic, hypoxic, nephrotoxic, septic, inflammatory, and obstructive forms. An improved understanding of the molecular underpinnings of AKI subtypes was ushered in two decades ago with the advent of transcriptome profiling technologies. Data mining of gene expression profiles from 150 microarray experiments performed in 21 different models of AKI (including mouse, rat, pig, and human models) identified novel upregulated genes that have now been well-characterized and are now considered “usual suspects” in AKI parlance—including *LCN2* (encoding lipocalin 2 or NGAL), *KIM-1* (kidney injury molecule-1), *CCL2* (chemokine ligand 2 or MCP-1), *HMOX1* (heme oxygenase), *TNF* (tumor necrosis factor), and *CLU* (Clusterin) (7). Downstream translational analyses in animal and human AKI are now beginning to yield pathways for therapeutic targeting, as well as excellent non-invasive assays for the early diagnosis of AKI and its sequelae (1).

More recent deep sequencing studies have identified significant differences in the responses between the AKI subtypes. For example, there exists a remarkable diversity of changes in the kidney genomic response to ischemic and septic injuries (8). While *TNF* and *LCN2* are dramatically upregulated in both ischemic and septic AKI, *KIM-1* is induced primarily in ischemic injury and *ICAM-1* in sepsis only (8). Furthermore, a comparison of the ischemic and the volume depletion models of AKI, often considered to be a continuum and therefore predicted to have similar gene expression response, unexpectedly showed that <10% of the expressed genes were differentially regulated in the two models despite identical elevations in serum creatinine (9). Volume depletion induced metabolic pathways and anti-inflammatory molecules. By contrast, ischemic injury activated hundreds of known and novel inflammatory, coagulation, and epithelial repair pathways, including the “usual suspects” *LCN2, KIM-1, CXCL1*, and *IL-6*, all of which were totally unchanged in the volume depletion model (9). For added complexity, different nephron segments responded with distinct signatures to different injuries. For example, volume depletion predominately affected the inner medulla, whereas ischemic changes were noted primarily in the outer medulla. In addition, ischemic injury induces mRNA expression of *KIM-1* specifically in the proximal tubule and, in contrast, *LCN2* specifically in the distal nephron (9). Hence, different insults lead to diverse responses reflecting alterations in segment-specific pathophysiology.

Recent metabolomic approaches have further validated additional dramatic differences in the response of the kidney to injuries that were previously thought to be closely related. For example, experimental models of ischemia–reperfusion injury display the rapid appearance of alanine, leucine, and glucose in urine, with a downregulation of urinary creatinine and nicotinamide (10). In marked contrast, hypoxic injury rapidly induces the urinary excretion of benzoate and fructose, while citrate and isothionate are suppressed (11). The differential appearance of these metabolites in the urine may hold important clues toward etiology-specific biomarkers and therapeutic targets in humans.

Additional recent metabolomic studies in a mouse model of ischemic AKI have identified a deficiency in the urinary and intra-renal nicotinamide adenine dinucleotide (NAD), an essential component of energy generation via glycolysis and the Kreb's cycle (12). In a phase I study of oral NAM supplementation (which generates NAD via a salvage pathway) in adults undergoing cardiac surgery, the rise in serum creatinine was prevented compared to placebo (12). Additional studies are underway.

## Strategies to Improve Bench-to-Bedside Translation in Aki

Thus, different etiologies of AKI elicit dramatically divergent responses. Additional basic and translational studies, too numerous to be elucidated here, have yielded characteristic structural, functional, and regenerative responses to each AKI stimulus. However, animal studies do not faithfully recapitulate the human AKI phenotype, rendering bench-to-bedside translation enormously challenging. Despite promising pre-clinical data, the human AKI literature is littered with numerous disappointing treatment failures—including forced diuresis and RGD peptides for tubular obstruction, ATP donors (ATP-Mg, thyroxine) for intracellular ATP depletion, natriuretic peptides and dopamine for vasoconstriction, reactive oxygen species scavengers and iron chelators for oxidative stress, anti-ICAM antibodies for endothelial–leucocyte adhesion, anti-apoptotic agents, growth factors (IGF-1, HGF, FGF, and erythropoietin), anti-inflammatory agents (α-MSH), and regenerative factors (mesenchymal stem cells) (13). The strategies proposed by the author to close this bench-to-bedside chasm are detailed below.

### Use Agnostic Approaches to Better Understand AKI in Animal and Human Models

We recommend the use of unbiased approaches to identify AKI susceptibility genes in humans. Large-scale genome-wide association studies (GWAS) can identify potentially pathogenic genomic sequences that are statistically enriched in AKI cases compared to controls. A recent GWAS analysis of a discovery cohort of 1,400 adults with critical illness (760 with AKI) followed by a separate replication cohort of 200 AKI cases (14) has yielded two single-nucleotide polymorphisms (SNPs) involving the transcription factor interferon regulatory factor 2 (IRF2) and an additional two SNPs close to the transcription factor T-box 1 (TBX1). The identification of SNPs near IRF2 suggests a potential role for the immune system in AKI, a concept with already strong biologic plausibility. TBX1 is expressed during kidney development, and this finding supports the intriguing concept that ontogeny recapitulates phylogeny after kidney injury, whereby genetic programs involved in nephrogenesis that become dormant after birth are once again reactivated and are essential for the recovery process after injury in post-natal life. Additional GWAS studies with even larger cohorts of control and AKI subjects are underway and may yield new AKI susceptibility genes of critical biological significance.

We recommend the employment of dramatic advances in single-cell RNA sequencing to examine both animal and human AKI models (15, 16). A recent comprehensive analysis of a mouse model of ischemia–reperfusion AKI using whole kidney total mRNA sequencing has already identified time-dependent changes in the expression of genes involved in tubular injury/repair, fibrosis, and innate and adaptive immunity (17). As an extension to humans, the NIH-funded Kidney Precision Medicine Project will analyze human AKI kidney biopsies based on elevations in serum creatinine and a urinary biomarker (based on the “usual suspects” previously mentioned). Single-cell RNA-seq and other advanced deep sequencing studies are expected to yield a detailed molecular atlas of the human kidney and potentially identify new pathways for future therapies.

### Establish Irrefutable Biologic Plausibility in Multiple Animal Models Prior to Embarking on Etiology-Specific Human Studies

We recommend detailed molecular analyses of animal models most pertinent to human AKI, followed by bioinformatic determination of both common and etiology-specific pathways, and downstream confirmation of biologic significance with additional techniques. Such studies will begin to address the enormous complexity of human AKI, which is often multifactorial, with overlapping components—in addition to volume depletion, ischemia–reperfusion injury, and nephrotoxins, clinicians have to worry about hypoxia, sepsis, inflammation, obstruction, and primary kidney diseases, to name a few situations. All of these induce comparable elevations in serum creatinine levels, the current highly flawed “gold standard” for the diagnosis and staging of AKI, a major limiting factor in AKI diagnostics today (1).

Recent publications validate this recommendation. Reliable animal models have now been developed to recapitulate many human AKI pathophysiologies, including the AKI-to-CKD transition (18). A careful analysis of these models has begun to elucidate the myriad responses at the structural and the molecular levels (17). In a murine bilateral ischemia–reperfusion survival model that recapitulated the human AKI-to-CKD transition, serum creatinine peaks after 2 days. However, histology at day 1 already revealed characteristic tubular changes in the outer medullary region that mimic the human phenotype. At 6 months after the injury, cortical fibrosis is the predominant finding. At 1 year later, additional cystic changes and a severe chronic interstitial nephritis appear, all reminiscent of end-stage kidney disease in humans. At the molecular level, the earliest changes included a significant expression of immediate early response and stress-related genes that are conserved between mouse and humans and are also activated soon after transplanting deceased donor kidneys (17). Within 24 h of injury, elevation of genes regulating apoptosis and proliferation, which persisted for weeks after injury, was prominently noted, attesting to the critical significance for the balance between cell death and cell survival during recovery from AKI. Most prominent among these were the genes encoding NGAL and KIM-1, both crucial to the processes of cellular regeneration and repair, lending ample biological plausibility for their roles as early non-invasive biomarkers (1, 7).

Animal models of ischemia–reperfusion AKI have additionally identified diverse epigenetic changes that in turn control AKI gene expression (8). For example, histone acetylation (which results in a transcriptionally permissive chromatin structure) was significantly associated with *Tnf* gene expression. Similarly, changes in histone methylation (which provides docking sites for chromatin modifiers) were identified in *Tnf*, *Kim-1*, and *Ngal* genes (8). These and other epigenetic changes are potentially reversible with appropriate pharmacotherapy and provide novel targets for drug design in AKI.

A translational molecular analysis of septic AKI has been limited by the fact that most animal models of this condition do not faithfully mimic the human condition (19). Murine models have utilized cecal ligation and puncture, fecal implantation in the abdomen, and lipopolysaccharide (LPS) injections and have resulted in varying degrees of structural and functional AKI. Despite these limitations, recent studies have revealed dramatic differences in gene transcription and in epigenetic changes in the kidney following LPS injection vs. ischemia–reperfusion (8). While the *Tnf* and *Ngal* genes are strongly induced in both models, *Kim-1* and *Tlr4* were upregulated only in ischemia–reperfusion and *Icam-1* only after sepsis. Many genes such as *Klotho* and *Netrin1* were downregulated only after ischemia–reperfusion, while several angiogenic genes exhibited a decreased expression following LPS injection. Understanding the heterogeneity of genetic and epigenetic responses in etiology-specific animal models will contribute to the discovery of interventions tailored to the cause of AKI.

### Identify Patients at Risk for AKI Pre-injury

We recommend the use of clinical scoring systems to predict AKI pre-injury. For example, to predict AKI in adults, clinical risk factors have been incorporated into the well-known Cleveland Clinic Score (for cardiac surgery) and Mehran Score (for contrast agents). These scores can be adapted for use in pediatrics. Indeed in a prospective multicenter analysis of children undergoing cardiac surgery, risk factors associated with a greater AKI incidence included lower age, weight, body surface area, and preoperative serum creatinine (20). Longer bypass time was also associated with AKI development. Those that had a bypass time >180 min showed a nearly 8-fold greater odds of developing AKI when compared to those with a bypass time <60 min (20).

We recommend the use of non-invasive pre-procedural biomarkers to predict AKI in children at risk. One of the best studied pre-operative biomarkers is uromodulin, a well-established nephroprotective protein—uromodulin knock-out mice are more susceptible to ischemia–reperfusion kidney injury and are more likely to experience tubular inflammation and necrosis, especially in the highly susceptible S3 segment of the proximal tubule (21). In a recent analysis of 101 children undergoing cardiac surgery with bypass, 47% developed AKI, and only 8% of the patients in the highest quartile of preoperative urinary uromodulin (uUMOD) concentrations developed AKI, in contrast with 92% of the participants in the lowest quartile (22). Preoperative uUMOD strongly predicted postoperative AKI, with area under the curve (AUC) of 0.90 (22). These results suggest that if pre-operative uUMOD is used to identify patients at risk for AKI after bypass, preventive measures might minimize post-operative AKI.

### Initiate Preventive Interventions Pre-injury in Susceptible Patients

We recommend the initiation of safe and inexpensive preventive measures in context-specific susceptible patients. One well-studied example is the use of N-acetyl cysteine (NAC) to prevent contrast-induced AKI. While this remains controversial, it is safe and orally effective. It is our practice to administer NAC (in addition to intravenous hydration and urinary alkalinization) in children scheduled for a contrast study with underlying chronic kidney disease stage 3 or greater who have a history of contrast-induced AKI (23).

Although intense renal vasoconstriction and vasospasm are well-known pathogenic processes in many forms of AKI, vasodilator therapies such as the natriuretic peptides and dopamine have been ineffective in preventing human AKI and are not recommended (23). However, the use of fenoldopam, a short-acting selective dopamine-1 receptor agonist (which the renal vasculature is particularly enriched in) with additional anti-inflammatory properties, is intriguing. In a prospective randomized double-blind trial of 80 children undergoing cardiac surgery, 40 received placebo and 40 were treated with fenoldopam intra-operatively (24). The fenoldopam group displayed a significant reduction in urinary biomarkers of AKI (NGAL and cystatin C) and a reduced need for diuretics and other vasodilators in the post-operative period. These data are promising but need independent confirmation in larger studies.

### Identify Patients Who Are at Risk for AKI Post-injury Early

We recommend the use of clinical scoring systems to predict AKI post-injury or when the timing of injury is unknown. The Renal Angina Index (RAI) has emerged as a useful scoring system in children who are critically ill (25). The RAI combines validated clinical risk factors (ICU admission, solid organ or stem cell transplant, mechanical ventilation, and vasopressor need) with evidence for decreased kidney function (increases in serum creatinine or degrees of fluid accumulation) to stratify patients at risk for subsequent severe AKI. Importantly, the incorporation of urinary biomarkers further improves the predictive ability of the RAI (26). In a prospective study of 184 children admitted to the pediatric ICU, a positive RAI (score ≥8) was present in 33% of patients at day 0 and predicted day 3 AKI with an AUC of 0.80. Inclusion of admission urinary NGAL further increased the AUC to predict day 3 AKI to 0.97. The RAI has thus emerged as an important tool to direct biomarker measurements in select patients who are most likely to benefit from such determinations.

We recommend the use of non-invasive biologically plausible urinary and plasma biomarkers to predict AKI and its severity in all clinical settings that portend a risk for AKI development. The most extensively studied such biomarker is NGAL, which is rapidly induced in the distal nephron following a variety of injurious stimuli and exerts a profound nephroprotective effect due to its anti-apoptotic, pro-proliferative, and bacteriostatic properties (1, 7, 27–29). A myriad of prospective studies, many in children, have now established the highly predictive role of NGAL as a biomarker to predict AKI and its complications in numerous clinical settings including critical illness, sepsis, cardiac surgery, nephrotoxins, and organ transplants (30–33). Following an analysis of many thousands of subjects and many thousands of AKI events in the literature, we now have six large meta-analyses attesting to the diagnostic properties of NGAL measurements for AKI prediction, with a consistent area under the curve of over 0.8 for a urinary NGAL value >150 ng/ml (34–38).

The combination of two biomarkers of cellular stress, namely, TIMP-2 and IGFBP-7, has also recently emerged as a promising early biomarker of AKI, particularly in adults with critical illness. In a recent meta-analysis of five studies with 1,619 critically ill patients, urinary TIMP-2 × IGFBP7 cutoff points of 0.3 (ng/ml)/1,000 had an AUC of 0.75 for AKI prediction (39). The pediatric experience with TIMP-2 and IGFBP-7 has been limited to date to small studies in which these biomarkers have been promising but are somewhat delayed predictors of AKI in comparison to NGAL (40, 41).

### Use Automated Electronic Health Records Plus Early Biomarkers to Assess for AKI

We recommend employing automated electronic health record (EHR) systems to direct measurements of serum creatinine and other predictive AKI biomarkers in children at risk for AKI. The utility of this approach is best exemplified by a prospective single-center study in which the EHR identified hospitalized children receiving aminoglycosides for more than 3 days or more than three nephrotoxins simultaneously (42). This triggered the pharmacists to recommend daily serum creatinine monitoring in exposed patients. During the study period, the exposure rate decreased by 38%, and the AKI rate decreased by 64%. An estimated 633 exposures and 398 AKI episodes were avoided. Thus, EHR-based surveillance for nephrotoxic medication exposure can lead to sustained reductions in nephrotoxin use and AKI. These interventions have now been translated to 30 other pediatric centers.

We recommend the use of the EHR-based serum creatinine increases to trigger biomarker measurements. In our clinical practice, urine NGAL is measured automatically in critically ill children with 50% or greater increase in serum creatinine. The results drive an early nephrology consultation as well as rational initial management, depending on the presence of intrinsic structural AKI (NGAL ≥ 150 ng/ml) or volume-responsive functional AKI (NGAL ≤ 50 ng/ml).

### Use Sequential Biomarkers to Initiate Context-Specific Therapies

**Figure 1:**
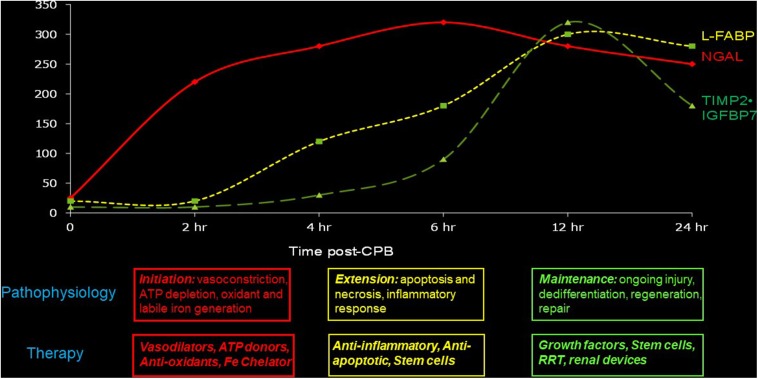
Sequential biomarkers to establish the time of initial injury as well as to initiate appropriate therapies based on knowledge of the underlying pathophysiology.

We recommend the use of temporally sequential biomarkers to establish the time of initial injury as well as to initiate appropriate therapies based on knowledge of the underlying pathophysiology. Experimental AKI proceeds in four sequential phases: initiation, extension, maintenance, and recovery. During the initiation phase, there is profound intracellular ATP depletion and generation of reactive oxygen molecules and labile iron. Vasodilator, ATP-donor, anti-oxidant, and iron chelation therapies may be effective during this phase, and the appearance of the earliest non-invasive biomarkers such as NGAL may be used to trigger such therapies. Several published studies in humans, including children with AKI (41, 43), have documented the appearance of NGAL in the urine and the blood very early after ischemic, nephrotoxic, or septic structural kidney injury (but not in pre-renal functional injury). In the extension phase, tubules undergo reperfusion-mediated cell death, and the injured endothelial and epithelial cells amplify the inflammatory cascades. This phase may be marked by intermediate biomarkers such as L-FABP, and therapeutic interventions might include anti-apoptotic and anti-inflammatory strategies. During the maintenance phase, cell regeneration predominates. Slightly delayed markers with high specificity, such as the cell cycle biomarkers (TIMP-2 and IGFBP-7), may trigger therapeutic measures such as growth factors and stem cells that accelerate repair. These concepts are illustrated in Figure 1 and are ready for implementation since both NGAL and the cell cycle biomarkers are now widely available for clinical use.

### Use Injury-Specific Biomarkers as Eligibility Criteria for Clinical Trial Enrollment

**Figure 2:**
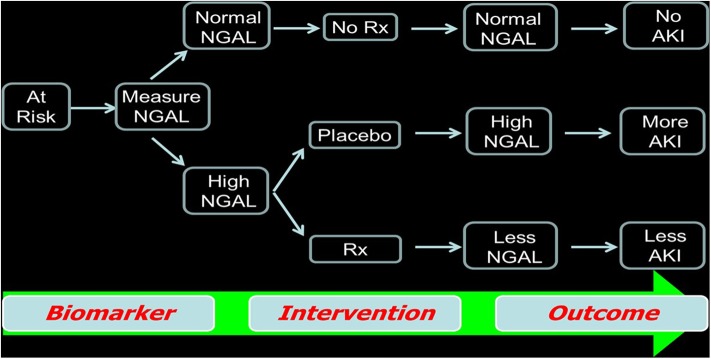
Use of biomarkers as criteria for enrollment in randomized clinical trials as well as to assess the efficacy of the agent being studied.

We recommend the use of widely available early AKI biomarkers such as NGAL to enroll patients in AKI clinical trials. This concept is illustrated in Figure 2, whereby patients known to have clinical risk factors for AKI are triaged using a biomarker measurement (irrespective of the serum creatinine value). Using early biomarker elevation to enroll subjects in AKI trials can increase the proportion of patients enrolled early in the course of AKI and decrease the sample size required, thereby dramatically reducing trial cost. This concept has been validated in recent publications using hypothetical simulations (44). In patients undergoing cardiac surgery, by using a combination of a known clinical risk factor (prolonged bypass time) and injury markers (IL-18 or NGAL), the authors showed that an AKI therapeutic trial cost could be decreased by 64% (44).

Furthermore, we recommend the use of AKI biomarkers to assess the response to therapies and as outcome measures to identify the therapies that warrant further testing in larger, multicenter trials. In this concept, a reduction in biomarker concentration can be considered as an initial success—this will once again decrease the cost of completing initial proof-of-principle AKI trials and will identify the best context-specific agents for more definitive trials that include widely accepted longer-term outcomes such as the “three Ds” (death, dialysis, and doubling of serum creatinine) and “MAKE” (major adverse kidney events).

### Use Injury-Specific Biomarkers in the Drug Development Process

We recommend the use of injury-specific biomarkers in the pre-clinical phases of drug development, specifically for the early identification of nephrotoxic AKI independent of the serum creatinine. Extensive pre-clinical studies by the academia and the industry have revealed highly specific urinary biomarkers that predict structural nephrotoxic AKI in the absence of serum creatinine increase. In 2008, the Food and Drug Administration (FDA) and the European Medicines Agency approved the use of seven safety biomarkers in pre-clinical drug toxicity, and this initial roster was extended in 2016 to include NGAL. Consequently, in 2018, a safety biomarker panel was approved by the FDA (including clusterin, cystatin C, KIM-1, NAG, NGAL, and osteopontin) to detect kidney tubular injury in healthy human volunteers participating in phase 1 clinical trials (45). It is hoped that the use of this safety biomarker panel can be extended to phases 2 and 3 AKI clinical trials, not only to identify any nephrotoxic injury or lack of therapeutic response (whereby biomarker concentration would increase) early but also to establish therapeutic efficacy (evidenced by a downward trajectory in biomarker concentration).

### Early Initiation of Anti-fibrotic Therapies

We recommend for clinical trials to investigate the early initiation of anti-fibrotic therapies. Recent publications validate this recommendation. Clinical evidence for the progression of pediatric AKI to CKD is now abundant. Animal models have now been developed to recapitulate many human AKI pathophysiologies, including the AKI-to-CKD transition (18). A careful analysis of these models has begun to challenge the dogma that the fibrotic response of the kidney to injury is a late and final common pathway. In a murine bilateral ischemia–reperfusion survival model that recapitulated the human AKI-to-CKD transition, the serum creatinine peaks after 2 days, and kidney sections at day 1 revealed surprising segment-specific responses. While significant acute tubular damage was noted in the outer medullary region, the same regions adjacent to the damaged S3 segments also unexpectedly revealed significant fibrosis (17). In the ensuing weeks, the outer medullary fibrosis extended further, with the additional appearance of cortical fibrosis.

Encouraging new experimental data suggest that this early fibrotic response can be prevented. In murine AKI due to ischemia–reperfusion, an intraperitoneal administration of a peptide (pUR4) that binds fibronectin and inhibits fibronectin polymerization (an early event in the fibrotic cascade) soon after injury dramatically attenuated the early fibrotic response (46). The pUR4 peptide was devoid of any adverse effects, rendering its translational application to human AKI a very realistic possibility.

## Conclusion

Propelled by the enabling technologies of molecular nephrology, this review has identified ten strategies that hold tremendous promise for effective bench-to-bedside translation to change the current dismal prognosis of pediatric AKI. These strategies are immediately actionable and well within the reach of the nephrology community. We are optimistic that this “call to arms” will be heeded, tested, and implemented.

## Author Contributions

PD is responsible for the concepts and contents of this publication.

### Conflict of Interest

PD is a co-inventor on patents submitted for the use of NGAL as a biomarker of kidney injury.

## References

[1] Desanti De Oliveira B Xu K Shen TH Callahan M Kiryluk K D'Agati VD. Molecular nephrologytypes of acute tubular injury. Nat Rev Nephrol. (2019) 15:599–612. 10.1038/s41581-019-0184-x 31439924 PMC7303545

[2] Potter SS. Single-cell RNA sequencing for the study of development, physiology and disease. Nat Rev Nephrol. (2018) 14:479–92. 10.1038/s41581-018-0021-7 29789704 PMC6070143

[3] Adam M Potter AS Potter SS. Psychrophilic proteases dramatically reduce single cell RNA-seq artifactsa molecular atlas of kidney development. Development. (2017) 144:3625–32. 10.1242/dev.151142 28851704 PMC5665481

[4] Chen L Lee JW Chou CL Nair AV Battistone MA Păunescu TG. Transcriptomes of major renal collecting duct cell types in mouse identified by single-cell RNA-seq. Proc Natl Acad Sci USA. (2017) 114:E9989–98. 10.1073/pnas.1710964114 29089413 PMC5699061

[5] Park J Shrestha R Qiu C Kondo A Huang S Werth M. Single-cell transcriptomics of the mouse kidney reveals potential cellular targets of kidney disease. Science. (2018) 360:758–63. 10.1126/science.aar2131 29622724 PMC6188645

[6] Wu H Malone AF Donnelly EL Kirita Y Uchimura K Ramakrishnan SM. Single-cell transcriptomics of a human kidney allograft biopsy specimen defines a diverse inflammatory response. J Am Soc Nephrol. (2018) 29:2069–80. 10.1681/ASN.2018020125 29980650 PMC6065085

[7] Devarajan P Genomic and proteomic characterization of acute kidney injury. Nephron. (2015) 13:185–91. 10.1159/000437237 PMC5650729 26491976

[8] Mar D Gharib SA Zager RA Johnson A Denisenko O Bomsztyk K. Heterogeneity of epigenetic changes at ischemia/reperfusion- and endotoxin-induced acute kidney injury genes. Kidney Int. (2015) 88:734–44. 10.1038/ki.2015.164 26061546 PMC4589440

[9] Xu K Rosenstiel P Paragas N Hinze C Gao X Huai Shen T. Unique transcriptional programs identify subtypes of AKI. J Am Soc Nephrol. (2017) 28:1729–40. 10.1681/ASN.2016090974 28028135 PMC5461802

[10] Chihanga T Ma Q Nicholson JD Ruby HN Edelmann RE Devarajan P. NMR spectroscopy and electron microscopy identification of metabolic and ultrastructural changes to the kidney following ischemia-reperfusion injury. Am J Physiol Renal Physiol. (2018) 314:F154–66. 10.1152/ajprenal.00363.2017 28978534 PMC5866452

[11] Chihanga T Ruby HN Ma Q Bashir S Devarajan P Kennedy MA. NMR-based urine metabolic profiling and immunohistochemistry analysis of nephron changes in a mouse model of hypoxia-induced acute kidney injury. Am J Physiol Renal Physiol. (2018) 315:F1159–73. 10.1152/ajprenal.00500.2017 29993280 PMC6230733

[12] Poyan Mehr A Tran MT Ralto KM Leaf DE Washco V Messmer J. *De novo* NAD^+^ biosynthetic impairment in acute kidney injury in humans. Nat Med. (2018) 24:1351–9. 10.1038/s41591-018-0138-z 30127395 PMC6129212

[13] Devarajan P. Update on mechanisms of ischemic acute kidney injury. J Am Soc Nephrol. (2006) 17:1503–20. 10.1681/ASN.2006010017 16707563

[14] Zhao B Lu Q Cheng Y Belcher JM Siew ED Leaf DE. A genome-wide association study to identify single-nucleotide polymorphisms for acute kidney injury. Am J Respir Crit Care Med. (2017) 195:482–90. 10.1164/rccm.201603-0518OC 27576016 PMC5378420

[15] Wu H Humphreys BD. The promise of single cell RNA-sequencing for kidney disease investigation. Kidney Int. (2017) 92:1334–42. 10.1016/j.kint.2017.06.033 28893418 PMC5696024

[16] Kiryluk K Bomback AS Cheng YL Xu K Camara PG Rabadan R. Precision medicine for acute kidney injury (AKI)Redefining AKI by agnostic kidney tissue interrogation and genetics. Semin Nephrol. (2018) 38:40–51. 10.1016/j.semnephrol.2017.09.006 29291761 PMC5753434

[17] Liu J Kumar S Dolzhenko E Alvarado GF Guo J Lu C. Molecular characterization of the transition from acute to chronic kidney injury following ischemia/reperfusion. JCI Insight. (2017) 2:94716. 10.1172/jci.insight.94716 28931758 PMC5612583

[18] Kumar S. Cellular and molecular pathways of renal repair after acute kidney injury. Kidney Int. (2018) 93:27–40. 10.1016/j.kint.2017.07.030 29291820

[19] Bellomo R Kellum JA Ronco C Wald R Martensson J Maiden M. Acute kidney injury in sepsis. Intensive Care Med. (2017) 43:816–28. 10.1007/s00134-017-4755-7 28364303

[20] Li S Krawczeski CD Zappitelli M Devarajan P Thiessen-Philbrook H Coca SG. Incidence, risk factors, and outcomes of acute kidney injury after pediatric cardiac surgerya prospective multicenter study. Crit Care Med. (2011) 39:1493–9. 10.1097/CCM.0b013e31821201d3 21336114 PMC3286600

[21] El-Achkar TM Wu XR Rauchman M McCracken R Kiefer S Dagher PC. Tamm-Horsfall protein protects the kidney from ischemic injury by decreasing inflammation and altering TLR4 expression. Am J Physiol Renal Physiol. (2008) 295:F534–44. 10.1152/ajprenal.00083.2008 18495803 PMC5504389

[22] Bennett MR Pyles O Ma Q Devarajan P. Preoperative levels of urinary uromodulin predict acute kidney injury after pediatric cardiopulmonary bypass surgery. Pediatr Nephrol. (2018) 33:521–6. 10.1007/s00467-017-3823-0 29058155 PMC5801051

[23] Devarajan P Prevention and management of acute kidney injury (Acute Renal Failure) in children. Available online at: https://www.uptodate.com (accessed Aug 1, 2019).

[24] Ricci Z Luciano R Favia I Garisto C Muraca M Morelli S High-dose fenoldopam reduces postoperative neutrophil gelatinase-associated lipocalin and cystatin C levels in pediatric cardiac surgery. Crit Care. (2011) 15:R160 10.1186/cc10295 21714857 PMC3219034

[25] Basu RK Kaddourah A Goldstein SL AWARE Study Investigators. Assessment of a renal angina index for prediction of severe acute kidney injury in critically ill childrena multicentre, multinational, prospective observational study. Lancet Child Adolesc Health. (2018) 2:112–20. 10.1016/S2352-4642(17)30181-5 30035208 PMC6053052

[26] Menon S Goldstein SL Mottes T Fei L Kaddourah A Terrell T. Urinary biomarker incorporation into the renal angina index early in intensive care unit admission optimizes acute kidney injury prediction in critically ill childrena prospective cohort study. Nephrol Dial Transplant. (2016) 31:586–94. 10.1093/ndt/gfv457 26908772 PMC6281075

[27] Mishra J Ma Q Prada A Mitsnefes M Zahedi K Yang J. Identification of neutrophil gelatinase-associated lipocalin as a novel early urinary biomarker for ischemic renal injury. J Am Soc Nephrol. (2003) 14:2534–43. 10.1097/01.asn.0000088027.54400.c6 14514731

[28] Mishra J Mori K Ma Q Kelly C Barasch J Devarajan P. Neutrophil gelatinase-associated lipocalina novel early urinary biomarker for cisplatin nephrotoxicity. Am J Nephrol. (2004) 24:307–15. 10.1159/000078452 15148457

[29] Mishra J Mori K Ma Q Kelly C Yang J Mitsnefes M. Amelioration of ischemic acute renal injury by neutrophil gelatinase-associated lipocalin. J Am Soc Nephrol. (2004) 15:3073–82. 10.1097/01.ASN.0000145013.44578.45 15579510

[30] Mishra J Dent C Tarabishi R Mitsnefes MM Ma Q Kelly C. Neutrophil gelatinase-associated lipocalin (NGAL) as a biomarker for acute renal injury after cardiac surgery. Lancet. (2005) 365:1231–8. 10.1016/S0140-6736(05)74811-X 15811456

[31] Hirsch R Dent C Pfriem H Allen J Beekman RH III Ma Q. NGAL is an early predictive biomarker of contrast-induced nephropathy in children. Pediatr Nephrol. (2007) 22:2089–95. 10.1007/s00467-007-0601-4 17874137

[32] Krawczeski CD Woo JG Wang Y Bennett MR Ma Q Devarajan P. Neutrophil gelatinase-associated lipocalin concentrations predict development of acute kidney injury in neonates and children after cardiopulmonary bypass. J Pediatr. (2011) 158:1009–15.e1. 10.1016/j.jpeds.2010.12.057 21300375

[33] Haase M Devarajan P Haase-Fielitz A Bellomo R Cruz DN Wagener G The outcome of neutrophil gelatinase-associated lipocalin-positive subclinical acute kidney injurya multicenter pooled analysis of prospective studies. J Am Coll Cardiol. (2011) 57:1752–61. 10.1016/j.jacc.2010.11.051 21511111 PMC4866647

[34] Haase M Bellomo R Devarajan P Schlattmann P Haase-Fielitz A NGAL Meta-analysis Investigator Group. Accuracy of neutrophil gelatinase-associated lipocalin (NGAL) in diagnosis and prognosis in acute kidney injurya systematic review and meta-analysis. Am J Kidney Dis. (2009) 54:1012–24. 10.1053/j.ajkd.2009.07.020 19850388

[35] Haase-Fielitz A Haase M Devarajan P. Neutrophil gelatinase-associated lipocalin as a biomarker of acute kidney injurya critical evaluation of current status. Ann Clin Biochem. (2014) 51:335–51. 10.1177/0004563214521795 24518531 PMC4104776

[36] Zhou F Luo Q Wang L Han L. Diagnostic value of neutrophil gelatinase-associated lipocalin for early diagnosis of cardiac surgery-associated acute kidney injurya meta-analysis. Eur J Cardiothorac Surg. (2016) 49:746–55. 10.1093/ejcts/ezv199 26094017

[37] Wang K Duan CY Wu J Liu Y Bei WJ Chen JY. Predictive value of neutrophil gelatinase-associated lipocalin for contrast-induced acute kidney injury after cardiac catheterizationa meta-analysis. Can J Cardiol. (2016) 32:1033.e19–29. 10.1016/j.cjca.2015.09.011 26860774

[38] Zhang A Cai Y Wang PF Qu JN Luo ZC Chen XD. Diagnosis and prognosis of neutrophil gelatinase-associated lipocalin for acute kidney injury with sepsisa systematic review and meta-analysis. Crit Care. (2016) 20:41. 10.1186/s13054-016-1212-x 26880194 PMC4754917

[39] Zhang D Yuan Y Guo L Wang Q. Comparison of urinary TIMP-2 and IGFBP7 cut-offs to predict acute kidney injury in critically ill patientsA PRISMA-compliant systematic review and meta-analysis. Medicine. (2019) 98:e16232. 10.1097/MD.0000000000016232 31261582 PMC6617439

[40] Gist KM Goldstein SL Wrona J Alten JA Basu RK Cooper DS. Kinetics of the cell cycle arrest biomarkers (TIMP-2^*^IGFBP-7) for prediction of acute kidney injury in infants after cardiac surgery. Pediatr Nephrol. (2017) 32:1611–9. 10.1007/s00467-017-3655-y 28382566

[41] Dong L Ma Q Bennett M Devarajan P. Urinary biomarkers of cell cycle arrest are delayed predictors of acute kidney injury after pediatric cardiopulmonary bypass. Pediatr Nephrol. (2017) 32:2351–60. 10.1007/s00467-017-3748-7 28755073 PMC7441589

[42] Goldstein SL Mottes T Simpson K Barclay C Muething S Haslam DB. A sustained quality improvement program reduces nephrotoxic medication-associated acute kidney injury. Kidney Int. (2016) 90:212–21. 10.1016/j.kint.2016.03.031 27217196

[43] Krawczeski CD Goldstein SL Woo JG Wang Y Piyaphanee N Ma Q. Temporal relationship and predictive value of urinary acute kidney injury biomarkers after pediatric cardiopulmonary bypass. J Am Coll Cardiol. (2011) 58:2301–9. 10.1016/j.jacc.2011.08.017 22093507 PMC3220882

[44] Parikh CR Moledina DG Coca SG Thiessen-Philbrook HR Garg AX. Application of new acute kidney injury biomarkers in human randomized controlled trials. Kidney Int. (2016) 89:1372–9. 10.1016/j.kint.2016.02.027 27165835 PMC4869991

[45] Leptak C Stockbridge N Qualification determination letter (Food and Drug Administration, 2018). Available online at:https://www.fda.gov/downloads/Drugs/DevelopmentApprovalProcess/DrugDevelopmentToolsQualificationProgram/BiomarkerQualificationProgram/UCM618888.pdf

[46] Bowers SLK Davis-Rodriguez S Thomas ZM Rudomanova V Bacon WC Beiersdorfer A. Inhibition of fibronectin polymerization alleviates kidney injury due to ischemia-reperfusion. Am J Physiol Renal Physiol. (2019) 316:F1293–8. 10.1152/ajprenal.00117.2019 31017009 PMC6620592

